# Diagnosis and Management of Congenital Dyserythropoietic Anaemia Type II in a Secundigravida

**DOI:** 10.7759/cureus.1811

**Published:** 2017-10-31

**Authors:** Anna Christoforidou, Zacharias Fasoulakis, Emmanuel N Kontomanolis

**Affiliations:** 1 Department of Hematology, Democritus University of Thrace, University Hospital of Alexandroupolis; 2 Obstetrics and Gynecology, Democritus University of Thrace, University Hospital of Alexandroupolis; 3 Obstetrics and Gynecology, Democritus University In Thrace, University Hospital of Alexandroupolis

**Keywords:** anisopoikilocytosis, splenomegaly, congenital dyserythropoietic anemia, homozygous missense mutation, sec23b gene, erythropoiesis

## Abstract

Congenital dyserythropoietic anaemias (CDAs) are very rare, heterogeneous hereditary red blood cell disorders characterized by ineffective erythropoiesis, erythroblast morphological abnormalities, haemolysis, and hypoglycosylation of red-blood-cell membrane proteins and lipids. There are four types (I-IV) of the disease identified, and all of them are associated with abnormal maturation and division of erythroid precursors. We report the management of a rare case of CDA type II diagnosed in a 26-year-old pregnant woman.

## Introduction

Congenital dyserythropoietic anaemia type II (CDA type II), or hereditary erythroblastic multinuclearity with positive acidified serum lysis test (HEMPAS), is an autosomal recessive disorder characterized by ineffective erythropoiesis. CDA type II is the most frequent congenital dyserythropoietic anaemia, caused by various homozygous or compound heterozygous mutations in the SEC23B gene, resulting in hypoglycosylation of the erythrocyte band three [1-2].

## Case presentation

A 26-year-old gravida 2, para 0 with increasing fatigue over the last month and a previous first-trimester miscarriage, visited the gynaecological medicine clinic at the sixth week of gestation. She had mild jaundice. Her medical history was significant for persistent anaemia since childhood, without a definite diagnosis other than “Gilbert Syndrome” despite investigation, and sustaining a haematocrit (Hct) between 29-30%, without transfusions. She was referred to the Haematology clinic. Her complete blood count (CBC) showed a hematocrit 29.6%, hemoglobin (Hb) 9.9 g/dl, mean corpuscular volume (MCV) 91 fl, red-blood-cell count (RBC) 2.4 mill/ml, white blood cell count (WBC) 5.9 x 10^9^/L (neutrophils 55%, lymphocytes 39%, monocytes 3%) and 171 x 10^9^/L platelets (PLT). Peripheral blood smear examination showed prominent anisopoikilocytosis, basophilic stippling, and the presence of erythroblasts. Serum chemistry showed high lactate dehydrogenase levels (LDH) at 231 IU/L and indirect bilirubin at 4.7 mg/dl. The direct Coombs test was negative, and serum B12, folic acid, and G-6PD levels were normal. Serum ferritin was 135 ng/mL and remained stable throughout pregnancy. Serum haptoglobin was very low (0.8 mg/dl) at repeated testing, compatible with a chronic haemolytic anaemia. Haemoglobin electrophoresis was normal. There was no haemoglobinuria. She did not report any episodes of acute jaundice in the past, or any dark urine (Table 1).

**Table 1:** Serum test results

Test Requested	Results	Normal Range	Units
Haematocrit	29-30	38-46♀	%
Haemoglobin	9.9	12.1-15.1♀	g/dl
MCV	91	80-94	fL
RBC	2.4	3.8-5.5	x10^12^/L
WBC Neutrophils Lymphocytes Monocytes	5.9	4-11	x10^9^/L
	55 39 3	50-70 20-40 2-6	%
LDH	231	152-225	IU/L
Indirect Bilirubin	4.7	0.1-0.8	mg/d
Ferritin	135	12-150	ng/mL
Haptoglobin	0.8	30-200	mg/dl

Antinuclear antibodies (ANA), rheumatoid factor, anticardiolipin antibodies and lupus anticoagulant were negative; C3, C4 and C-reactive protein were within normal limits. Osmotic fragility test was normal. Abdominal ultrasound revealed mild splenomegaly and a chololithiasis, while the obstetrics ultrasonography was normal. Based on the peripheral blood findings, the case history of chronic haemolytic anaemia since childhood, and the inadequate reticulocyte response, our differential diagnosis included a congenital dyserythropoietic anaemia. However, the patient was reluctant to undergo a bone marrow aspiration and biopsy. As a result, we decided to put diagnosis on hold until delivery and observed the patient closely with transfusions as needed.

Oral iron supplements were strictly avoided. During pregnancy, she was transfused with two units of packed red blood cells (PRBC) in total in order to maintain a haemoglobin level greater than 7g/dl. A healthy male infant, weighing 3650gr, was delivered via Cesarean section at the 36th week of gestation.

**Figure 1:**
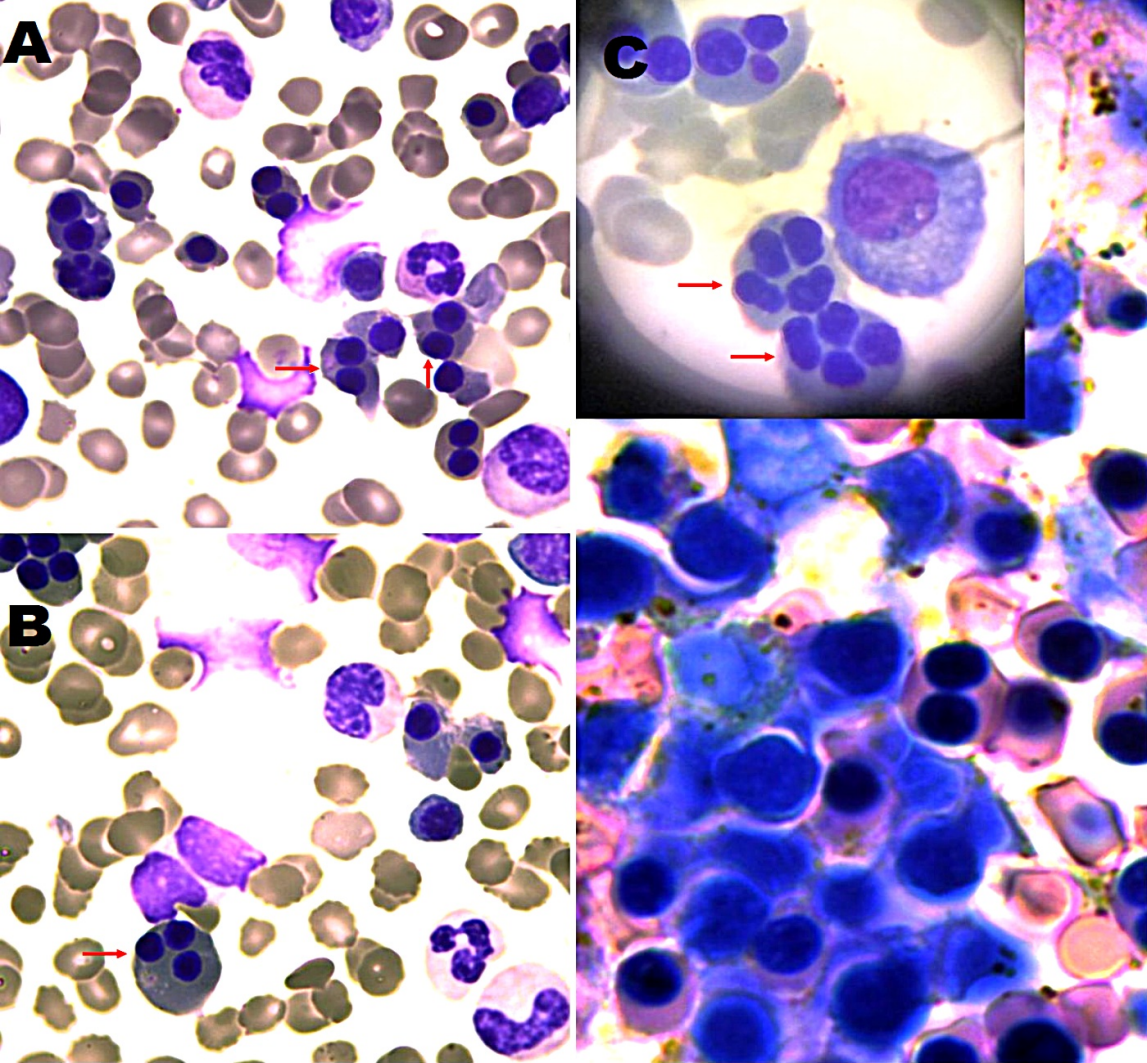
Bone marrow biopsy sections stained with Giemsa A: Section showing binucleated erythroblasts (indicated by red arrows). B and C: These sections show some erythroblasts with three and four nuclei (indicated by red arrows). These are not specific for CDA as they can be seen in other anaemias too (such as myelodysplastic syndromes), but in young patients with chronic haemolytic anaemia and without changes in white blood cells or platelets, they point directly to the diagnosis of CDA II.

Bone marrow biopsy was performed post-partum, showing marked erythroid hyperplasia with binuclear and multinuclear erythroblasts as well as increased haemosiderin deposits by Prussian blue stain (Figure 1).

In CDA II, more than 10% of the erythroblasts contain two equal nuclei, especially late erythroblasts. Other findings include a small percentage of multinucleated erythroblasts, abnormal chromatin structure, and karyorrhexis [3].

Direct sequencing of the SEC23B gene revealed a homozygous missense mutation in the exon 4 (c.325G > A), a mutation that leads to a variant protein with substitution of glutamic acid 109 by lysine (p.Glu109Lys) and has been described as a causative mutation in CDA II [4]. It also revealed 11 single nucleotide polymorphisms (SNPs) in various introns, as well as in the 5' untranslated region (5’UTR), whose causality has not yet been described. However, due to the lack of specimens from the patient’s parents, a more in-depth study of these SNPs could not be conducted. Based on these findings, the patient was diagnosed with congenital dyserythropoietic anaemia type II.

Weekly fetal ultrasound examination and adequate surveillance led to a safe delivery of a healthy baby without any complications.

## Discussion

The hallmark of the disease is the presence of binucleated erythroblasts in bone marrow. Like any other dyserythropoietic anaemia, it is associated with marked increase in dietary iron absorption and progressive iron overload with a suppressed serum hepcidin, which is not evident until symptoms of secondary haemochromatosis occur, affecting the liver, heart, pancreas and other organs. Evidently, haemosiderosis occurs even in non-transfusion dependent patients. Because of menstruation, females develop haemochromatosis later than males, usually post menopause. Other complications include splenomegaly and gallstones. Anaemia is usually mild, and many patients do not require transfusions, except for pregnancy and in case of infections. Less frequently intense extramedullary haemopoiesis can lead to the development of paravertebral masses. Diagnosis is made by the characteristic findings of peripheral blood and bone marrow, the positive acid haemolysis test (HAM test), sodium dodecyl sulfate polyacrylamide gel electrophoresis (SDS-PAGE) electrophoresis for the analysis of red blood cell membrane protein glycosylation, and the SEC23B gene sequencing. If the latter method is available, then SDS-PAGE can be omitted. The acid haemolysis test has largely been abandoned by most laboratories. The differential diagnosis includes other haemolytic anaemias such as congenital spherocytic anaemia, paroxysmal nocturnal hemoglobinuria, chronic non-spherocytic haemolytic anaemia due to G-6PD deficiency, autoimmune haemolytic anaemia, and anaemias with ineffective erythropoiesis like thalassemia and myelodysplastic syndromes. Congenital spherocytic anaemia - the most common misdiagnosis - is easily distinguished by the absence of anisopoikilocytosis and the higher percentage of reticulocytes, in advance of more specific tests such as osmotic fragility and protein electrophoresis [3].

## Conclusions

The management of CDA consists of transfusions when the haemoglobin decreases below 7 g/dl, or according to clinical symptoms and early prophylaxis from iron overload by phlebotomy, or iron chelating agents. Transfusion-dependent patients or those with massive splenomegaly may benefit from splenectomy. Allogeneic haematopoietic stem transplantation has been successfully used in three severely anaemic patients.

Pregnancy can aggravate anaemia, like in our patient, and increase the need for transfusions. This case underlines the important role of a correct diagnosis of a haemolytic anaemia being present in pregnancy. Iron supplementation, which is usually prescribed for pregnant women, would have been disastrous for this patient. In addition, the proper close follow-up with transfusions as needed may prevent the effects of severe anaemia, like the low birth weight or immaturity. This diagnosis, however very rare, can easily be reached with the aid of a haematologist in a tertiary centre.
